# Editorial: Minority Adolescent Mental Health and Health Care Disparities

**DOI:** 10.3389/fpubh.2021.704765

**Published:** 2021-06-18

**Authors:** Wenhua Lu, Lei Xu, Daniel Hart

**Affiliations:** ^1^Department of Community Health and Social Medicine, School of Medicine, City University of New York, New York, NY, United States; ^2^Department of Health Education and Promotion, College of Health and Human Performance, East Carolina University, Greenville, NC, United States; ^3^Department of Psychology, School of Arts and Sciences, Rutgers, The State University of New Jersey, Camden, NJ, United States

**Keywords:** adolescent mental health, healthcare disparities, immigrant health, multicultural stressors, immigrant paradox, machine learning technique

Mental health problems, including anxiety, mood, attention, and disruptive behavior disorders, continue to be severe public health concerns among adolescents. Globally, 1 in every 6 adolescents aged 10–19 experience a mental health problem (1); in the U.S., mental health problems are impacting up to 50% of adolescents in their lifetime (2, 3). Based on data from the National Survey on Drug Use and Health, for example, the prevalence of past-year major depression increased steadily from 8.3% in 2011 to 15.8% in 2019 among US adolescents aged 12–17 (4). Although effective treatments exist for many mental health problems, over half of adolescents in the U.S. who need mental health treatment never receive it (5). Further, compared with Whites, racial/ethnic minority adolescents in the U.S. are more vulnerable to mental health problems but less likely to use mental health services (6). In this Research Topic, we bring together a diverse panel of researchers to provide interdisciplinary perspectives to the topic of adolescent mental health and healthcare disparities in the U.S.

Specifically, in their systematic review, Lu et al. summarized existing literature on barriers and facilitators of mental health service use among racial/ethnic minority adolescents in the U.S., including Blacks/African Americans, Hispanics/Latinos, and Asian Americans. Using the socio-ecological framework, researchers identified a plethora of factors that can influence minority adolescents' access, attendance, and adherence to mental health treatment, including symptom severity and internal asset at the adolescent level, fears, etiological beliefs, and parenting issues at the parent level, patient-therapist ethnic match and relationship at the service provider level, acculturation at the cultural level, and social network support and school asset at the social level. Another important finding is the lack of attempt in literature to delineate any within-group differences among racial/ethnic subgroups.

Children of immigrants are often considered to be at increased risk of mental health problems due to families' immigration-related stress and perceived discrimination and prejudice from the host country. However, many studies found them to have better developmental outcomes than children with native-born parents in the U.S. In their study, Zhang et al. attempted to unfold such immigrant paradox by examining the mediating effect of child maltreatment risk in the association of parental nativity status and race/ethnicity with children's mental health. Findings from this study shed light on future research to further clarify the mechanism underlying different parenting practices between same race/ethnicity immigrants and native-born families so that culturally responsive interventions can be developed to protect minority children's mental health.

Increasingly, machine learning techniques have been applied in psychology to identify individuals with mental health conditions. In their pioneering work, Chiu et al. used a modern supervised learning method to build a predictive model to detect adolescents with depression and related severe functional impairment. Specifically, using a logistic model as a classifier (i.e., a predictive mechanism), the researchers successfully identified 66% of adolescents with major depression-related severe functional impairment from the pooled sample of adolescents in the National Survey on Drug Use and Health 2011–2017 with acceptable recall and reasonable accuracy rates. The algorithmic identification of adolescents at risk for depression and related severe functional impairment hold significant implications for prevention and early treatment of adolescent depression.

Adolescents in multicultural families are exposed to numerous stressors and face environmental vulnerability within the family, school, and community systems, which may affect their health and well-being. In their exploratory study, Shin et al. investigated social-cultural and community factors that may affect subjective well-being of adolescents from 206 multicultural families living in South Korea, i.e., families consisting of a marriage immigrant or foreigner with Korean citizenship. Multiple risk factors were identified, including weak social network support system, socioeconomic vulnerabilities of multicultural families, and acculturative stress. Findings from this study can help facilitate the development of multicultural support programs to improve well-being of adolescents in multicultural families in South Korea and also inform research inquiries with Korean American adolescents living in the U.S.

In conclusion, studies included in this Research Topic identify existing gaps in adolescent mental health research and call for continued efforts to eliminate disparities in mental healthcare services for racial/ethnic minority adolescents in the U.S.

## Author Contributions

WL drafted the Editorial. LX and DH revised and approved the final version. All authors contributed to the article and approved the submitted version.

## Conflict of Interest

The authors declare that the research was conducted in the absence of any commercial or financial relationships that could be construed as a potential conflict of interest.
